# Genetic Variants in the Promoter Region of the Macrophage Migration Inhibitory Factor are Associated with the Severity of Hepatitis C Virus-Induced Liver Fibrosis

**DOI:** 10.3390/ijms20153753

**Published:** 2019-07-31

**Authors:** Theresa Hildegard Wirtz, Petra Fischer, Christina Backhaus, Irina Bergmann, Elisa Fabiana Brandt, Daniel Heinrichs, Maria Teresa Koenen, Kai Markus Schneider, Thomas Eggermann, Ingo Kurth, Christian Stoppe, Jürgen Bernhagen, Tony Bruns, Janett Fischer, Thomas Berg, Christian Trautwein, Marie-Luise Berres

**Affiliations:** 1 Department of Internal Medicine III, RWTH Aachen University Hospital, 52074 Aachen, Germany; 2 Institute of Human Genetics, RWTH Aachen University Hospital, 52074 Aachen, Germany; 3 Department of Intensive Care Medicine, RWTH Aachen University Hospital, 52074 Aachen, Germany; 4 Chair of Vacular Biology, Institute for Stroke and Dementia Research (ISD), Ludwig-Maximilians-University (LMU), 81377 Munich, Germany; 5 Munich Cluster for Systems Neurology (EXC 2145 SyNergy), 81377 Munich, Germany; 6 Section of Hepatology, Clinic for Gastroenterology, University Hospital Leipzig, 04103 Leipzig, Germany

**Keywords:** HCV, liver fibrosis, macrophage migration inhibitory factor, promoter polymorphisms, hepatocellular carcinoma, biomarker

## Abstract

Two polymorphisms in the promoter region of macrophage migration inhibitory factor (MIF)—rs755622 and rs5844572—exhibit prognostic relevance in inflammatory diseases. The aim of this study was to investigate a correlation between these MIF promoter polymorphisms and the severity of hepatitis C virus (HCV)-induced liver fibrosis. Our analysis included two independent patient cohorts with HCV-induced liver fibrosis (504 and 443 patients, respectively). The genotype of the single nucleotide polymorphism (SNP) -173 G/C and the repeat number of the microsatellite polymorphism -794 CATT_5–8_ were determined in DNA samples and correlated with fibrosis severity. In the first cohort, homozygous carriers of the C allele in the rs755622 had lower fibrosis stages compared to heterozygous carriers or wild types (1.25 vs. 2.0 vs. 2.0; *p* = 0.03). Additionally, ≥7 microsatellite repeats were associated with lower fibrosis stages (<F2) (*p* = 0.04). Comparable tendencies were observed in the second independent cohort, where fibrosis was assessed using transient elastography. However, once cirrhosis had been established, the C/C genotype and higher microsatellite repeats correlated with impaired liver function and a higher prevalence of hepatocellular carcinoma. Our study demonstrates that specific MIF polymorphisms are associated with disease severity and complications of HCV-induced fibrosis in a stage- and context-dependent manner.

## 1. Introduction

Hepatitis C virus (HCV) infection is one of the most common causes of acute and chronic liver disease across the world. In approximately 75%–85% of patients with acute HCV infection, the virus persists and chronic hepatitis develops, which is characterized by a persistent intrahepatic inflammation contributing to liver fibrosis and liver damage [1]. However, only about 15% of infected patients will progress to cirrhosis within the first 20 years [2]. Molecular mechanisms of viral persistence and risk factors to develop advanced liver disease and hepatocellular carcinoma (HCC) have been in the focus of research for the last few decades. Numerous studies identified risk factors for accelerated fibrosis progression, such as intrahepatic inflammation (grading) and fibrosis (staging) on a baseline liver biopsy or infection with HCV genotype 3 as opposed to other genotypes [2,3,4].

Antiviral treatment of chronic HCV infection aims at preventing liver disease progression by achieving a sustained virological response (SVR). Fortunately, interferon-free treatment options with direct antiviral agents (DAA) have become available but impose high costs for healthcare systems in industrialized countries and are limited to patients with access to specialist care [5]. Predicting the intraindividual course of disease, even during the present era of DAA-therapy, could help in delaying and narrowing the indication of antiviral treatments in patients with chronic hepatitis C who are not at risk for liver fibrosis or extrahepatic manifestations. Furthermore, recent studies show that in spite of achieving SVR after DAA-therapy, approximately 10% of patients continue to have ongoing liver injury and may develop hepatic steatosis, implicating the necessity for long-term surveillance, even after SVR [6,7,8]. Therefore, the identification of genetic variants may help in estimating the intraindividual course of disease in untreated and successfully treated patients, and to individualize clinical monitoring.

Macrophage migration inhibitory factor (MIF) is a pro-inflammatory cytokine with chemokine-like functions and is known to be an important regulator of the innate and adaptive immune system [9,10]. MIF modulates various inflammatory and immune responses, which has prompted the characterization of MIF as a biomarker in several chronic inflammatory diseases, such as rheumatoid arthritis, systemic sclerosis, and inflammatory bowel disease [11,12,13]. Concerning chronic liver diseases, recent studies identified MIF as a mediator of chemokine production and immune cell infiltration in alcohol-induced liver steatosis [14]. MIF is also associated with the disease severity in autoimmune hepatitis and in cholestatic liver disease [15,16].

The human *MIF* gene is located on the long arm of chromosome 22 at position 22q11.23. Two polymorphisms in the promoter region of the *MIF* gene, rs755622 and rs5844572, have been found to be associated with the severity of inflammatory and autoimmune diseases including autoimmune hepatitis [15,17,18]. Both polymorphisms have been shown to be functionally relevant as they influence the gene expression and serum concentrations of MIF, where the rs755622 is a single nucleotide polymorphism (SNP). In the worldwide population, the genotype frequency of the C/C genotype ranges between 2% and 10% [19,20]. The rs5844572 is a tetra nucleotide microsatellite repetition of the tetra nucelotide “CATT” with a total repeat length ranging from five to eight repetitions resulting in the allelic variants CATT 5, CATT 6, CATT 7, or CATT 8. The basal transcriptional activity of the *MIF* promoter is increased in patients with higher repeats (CATT 7/X, 8/X) resulting in higher MIF serum levels [17]. In the general population, the allelic frequency of the CATT 6 allele is up to 50%, whereas higher repeat numbers are rare (allelic frequency of the CATT7 allele: 10%–20%) [21,22]. The -173 C allele is associated with increased gene expression and serum MIF levels most likely due to linkage disequilibrium with the -794 CATT 7 high expression allele [11,23].

Our study aimed at investigating the prognostic significance and clinical relevance of the two *MIF* promoter polymorphisms rs755622 and rs5844572 in patients with HCV-induced chronic liver disease.

## 2. Results

### 2.1. Cohort Characteristics of the HCV 2008 Cohort

Our study included two independent patient cohorts with HCV-induced liver fibrosis. For purposes of clarity, in the following paragraphs the first cohort will be named as the “HCV 2008 cohort,” and the second cohort will be named as the “HCV 2018 cohort.”

The cohort characteristics of the HCV 2008 cohort are presented in Table 1. Overall, 504 patients with chronic HCV-infection underwent liver biopsy before induction of treatment. Of these, 473 patients showed a Metavir staging of ≥F0.5 and were included in the study (Table 1). The median age was 54 years at the time of diagnosis of chronic hepatitis C infection. Fibrosis stage (staging) as well as intrahepatic inflammatory activity (grading) was analyzed using a histological assessment performed by experienced pathologists according to the Metavir score [24]. Approximately one quarter of all patients showed fibrosis stages < F1 or ≥ F3. More than three quarters of all patients showed mean fibrosis stages of F1–F1.5 or F2–F2.5. Eight percent of patients fulfilled the histological criteria of cirrhosis (fibrosis stage 4). Based on former treatment guidelines, patients received treatment with (pegylated) interferon mono, occasionally in combination with ribavirin and/or a protease inhibitor boceprevir or telaprevir (data not shown) after the baseline biopsy. Liver function parameters were not available in this cohort.

### 2.2. Genotype Frequency of -173 G/C SNP and -794 CATT_5–8_ Microsatellite in the HCV 2008 Cohort 

Allelic discrimination for rs755622 was successful in 471 (>99%) out of 473 patients with established fibrosis (stage > F0). No deviation from the Hardy–Weinberg equilibrium was observed (*p* = 0.56). Overall, two thirds (66%) of patients were identified to be “G/G.” Only 3% of all patients included showed homozygosity for the “C”-allele in the -173 G/C SNP. The minor allele frequency (0.18) was comparable to other European cohorts [20]. When stratified for the presence of significant fibrosis (fibrosis stage ≥ F2), the minor allele frequency of rs755622 was higher in patients with lower fibrosis (0.20 vs. 0.16; *p* = 0.014) (Table 2).

A total of 794 CATT_5–8_ microsatellite repeats were successfully discriminated in 468 (99%) out of 473 patients with established fibrosis (stage > F0). Overall, 24% of patients had seven or more microsatellite repeats on at least one allele (CATT 7X, CATT 8/X), which was comparable to other European populations [20]. Patients with significant fibrosis (≥F2) presented with high microsatellite repeats (CATT 7/X or 8/X) less often than patients without significant fibrosis (<F2) (21% vs. 30%; *p* = 0.04) (Table 3).

### 2.3. The G/C and C/C Genotypes in the -173 G/C SNP and High Microsatellite Repeat Numbers at -794 CATT_5–8_ Significantly Correlate with Lower Fibrosis Stages Independent of Inflammatory Activity in the HCV 2008 Cohort

**Figure 1:**
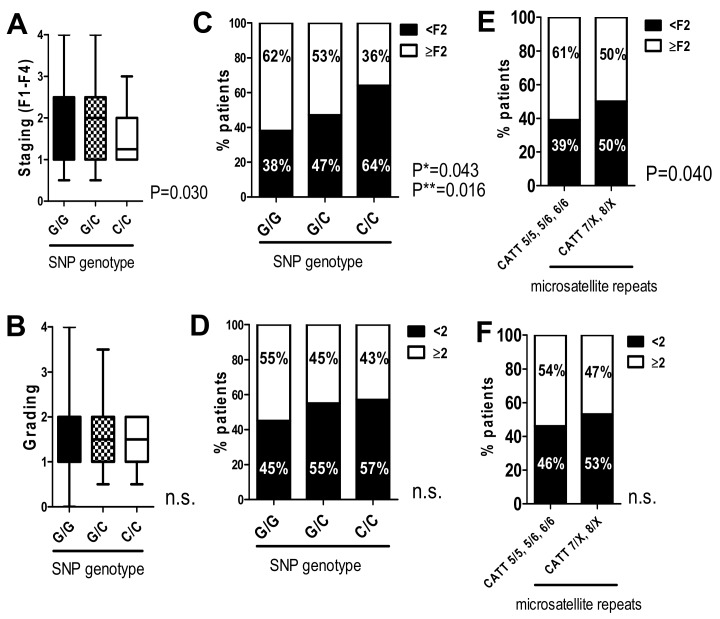
The C/C genotype in the -173 G/C-SNP and higher microsatellite repeat numbers in the CATT_5–8_ microsatellite significantly correlated with lower fibrosis stages in HCV-induced liver fibrosis. Only patients with fibrosis stage ≥F0.5 were included. (**A**) Correlation of the SNP genotype and fibrosis staging F1–F4 showed a significantly lower median fibrosis stage in patients with a C/C genotype compared to those with a C/G genotype (1.25 vs. 2.0) and G/G genotype (1.25 vs. 2.0, *p* = 0.030 in the Kruskal–Wallis test). (**B**) Histological grading did not significantly differ between the SNP-genotypes. (**C**) Proportions of patients with low or high fibrosis stages (<F2 vs. ≥F2) within the three SNP genotype subgroups. In the C/C subgroup, significantly more patients showed lower levels of fibrosis as Fisher´s exact test (*; *p* = 0.043) and linear-by-linear association test (**; *p* = 0.016) shows. (**D**) Histological grading did not significantly differ between the SNP-genotypes. (**E**) High repeat numbers on at least one allele of the microsatellite (“CATT 7/X; 8/X”) significantly correlated with lower fibrosis stages (*p* = 0.040). (**F**) There was no significant difference in the inflammatory activity of patients with low (“CATT 5/5, 5/6, 6/6”) to high repeat numbers. Data are expressed as the median ± range (**A**,**B**) or contingency (**C**,**D**–**F**) and were considered significant if *p* < 0.05 (*).

To investigate a clinical relevance of the two MIF promoter polymorphisms in more detail, the associations of the -173 G/C SNP and the microsatellite repeats with clinical and histological parameters were further investigated. Concerning the SNP (rs755622), the different genotypes G/G, G/C and C/C were correlated with fibrosis stages based on histological evaluation in these patient subgroups (Figure 1A,C). Patients carrying the C/C genotype had significantly lower fibrosis stages (median fibrosis stage F1.25) compared to patients with the G/C or G/G genotype (both had median fibrosis stage F2.0; *p* = 0.03 in the Kruskal–Wallis test, Figure 1A). This result was further confirmed using Fisher´s exact test (*p* = 0.043) and a linear-by-linear association test (*p* = 0.016, Figure 1C). The observed effect was independent of intrahepatic inflammation, as grading did not significantly differ between the genotypes (Figure 1B,D).

Investigating the microsatellite polymorphism revealed that patients with high microsatellite repeats on at least one allele had lower fibrosis stages (Figure 1E): 50% of patients with genotype CATT7/X or CATT8/X had fibrosis stages of F2 fibrosis or higher, whereas 61% of patients with lower repeat numbers (CATT 5/5, 5/6, 6/6) had higher fibrosis stages (*p* = 0.040). This effect was independent of intrahepatic inflammation, as grading did not differ between patients with different genotypes (Figure 1F). Together, these data demonstrate that the genotype of both MIF promoter polymorphisms, which are assumed to be associated with higher MIF serum levels, significantly correlated with fibrosis severity in this cohort of HCV-infected patients independent of the intrahepatic inflammatory status.

### 2.4. Cohort Characteristics of the HCV 2018 Cohort

To validate in an independent cohort, 443 patients with HCV-induced liver fibrosis were analyzed. Patients for the validation cohort (“HCV 2018 cohort”) were included in the study from 2010 to 2018. In contrast to the HCV 2008 cohort, fibrosis was determined using non-invasive transient elastography (TE), and the inflammatory activity was evaluated using serum transaminase levels. Baseline characteristics of the HCV 2018 cohort are presented in Table 4 with a median age of 52 years at the time of study inclusion. Sexes were well-balanced. Approximately one third of patients showed no liver fibrosis (TE < 6 kPa), increased liver stiffness according to liver fibrosis (TE 612 kPa) or liver cirrhosis (TE ≥ 12 kPa). The majority of patients with liver cirrhosis had Child–Pugh A compensated liver cirrhosis (76%). Eleven percent of patients presented with HCC. HCV genotype 1 was the most frequent genotype (83%). Treatment was initiated using DAAs after exclusion of the contraindications (data not shown).

### 2.5. Genotype Frequency of -173 G/C SNP and -794 CATT_5–8_ Microsatellite in the HCV 2018 Cohort

Allelic discrimination for rs755622 was successful in 433 out of 443 patients. Overall, only 3% of patients were identified to be homozygous for the “C” allele in the -173 G/C SNP position of the MIF promoter as expected compared to other European populations [20]. Approximately two thirds of patients (69%) were identified to be “G/G” (Table 5). The minor allele frequency was 0.17, and no deviation from the Hardy–Weinberg equilibrium was observed (*p* = 0.39). Transient elastography (TE) was available from 377 (87%) patients. Applying the cut-off for advanced fibrosis (TE ≥ 9 kPa), we observed no significant differences in allele frequencies between both groups (Table 5).

794 CATT_5–8_ microsatellite repeats were successfully discriminated in 348 patients. Similar to the 2008 cohort, in the 2018 cohort, 24% of patients had higher microsatellite repeats on at least one allele (CATT 7/X, 8/X) without differences between patients with and without advanced fibrosis (Table 6).

There were no significant differences in the prevalence of HCV genotype 1, 3, or “others,” including HCV genotype 2 and 4 between the SNP and microsatellite genotype subgroups in this HCV 2018 cohort (Supplementary Figure S1A,B).

### 2.6. The C/C Genotype in the -173 G/C SNP and High Microsatellite Repeat Counts in the -794 CATT_5–8_ Showed a Tendency Toward Lower Liver Stiffness Measurements in the HCV 2018 Cohort

**Figure 2:**
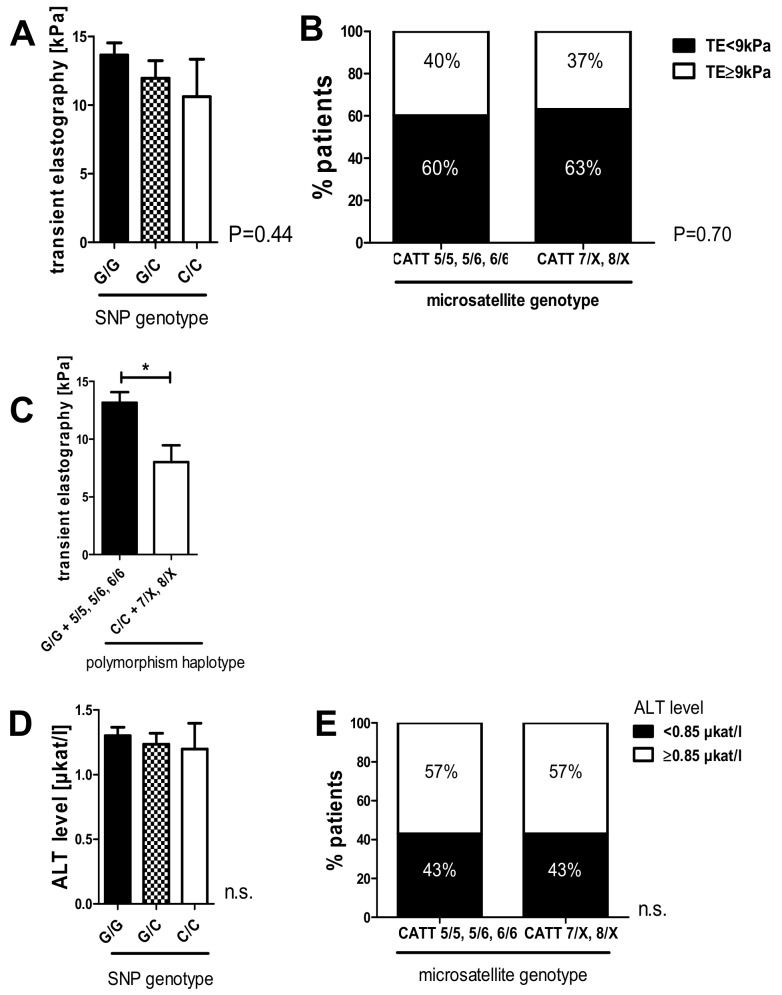
The C/C genotype of the -173 G/C SNP and higher microsatellite repeat numbers in the CATT_5–8_ microsatellite correlated with lower fibrosis stages in HCV-induced liver fibrosis in the HCV 2018 validation cohort. (**A**) Correlation of the SNP genotype and transient elastography (TE) results showed a tendency toward lower mean fibrosis measurements in patients with the C/C genotype compared the G/G genotype (*p* = 0.40). (**B**) High repeat numbers on at least one allele of the microsatellite (“CATT 7/X; 8/X”) did not significantly correlate with lower liver stiffness measurements. (**C**) The haplotype analysis of the G/G SNP genotype and low repeat numbers in the microsatellite (CATT 5/5, 5/6, 6/6) showed higher mean TE values than the C/C SNP genotype and high microsatellite repeat numbers (CATT 7/X, 8/X; *p* = 0.015). (**D**,**E**) Grading of the inflammatory activity based on levels of alaninaminotransferase (ALT) did not differ significantly between the SNP or microsatellite genotypes (patients were subgrouped by the ALT cutoff of 0.85 µkat/L). Data are expressed as the mean fibrosis level (**A**,**C**,**D**) or contingency (**B**,**E**) and were considered significant if *p* < 0.05 (*).

To validate our results from the HCV 2008 cohort, we correlated the rs755622 and rs5844572 polymorphisms with liver stiffness based on transient elastography (TE) and hepatic inflammation. Patients who were homozygous for the C-allele at -173 had a lower mean liver stiffness as measured using transient elastography compared to patients with G/G (10.6 kPa vs. 13.6 kPa) without reaching the level of statistical significance (Figure 2A, *p* = 0.44). Likewise, in the subgroup of patients with higher microsatellite repeat numbers (CATT 7/X, 8/X), 37% had advanced fibrosis using a TE cut-off of 9 kPa, as compared to 40% of patients with lower repeat numbers (CATT 5/5, 5/6, 6/6; Figure 2B; *p* = 0.70). We furthermore conducted a haplotype analysis (Figure 2C). Here, patients who showed homozygosity for the G-allele in the -173 G/C SNP, in addition to low repeat numbers in the microsatellite (G/G + CATT 5/5; 5/6 or 6/6), were revealed to have more advanced liver stiffness (mean TE value 13.14 kPa) compared to patients with a minor allele haplotype (C/C + 7/X, 8/X; mean TE value 8.02 kPa). This effect was statistically significant (*p* = 0.015).

As observed in the derivation cohort, the prognostic advantage of the minor alleles in both polymorphisms were not due to a lower grade of intrahepatic inflammation, as the comparison of G/G, G/C, and C/C genotypes in the SNP, as well as low and high repeat numbers in the microsatellite, did not show significant differences in alaninaminotransferase (ALT) levels (Figure 2 D,E).

### 2.7. The G/C and C/C Genotypes in the -173 G/C SNP and High Microsatellite Repeat Counts in the -794 CATT_5-8_ Were Associated with Worse Liver Function in Patients with HCV-Induced Liver Cirrhosis

**Figure 3:**
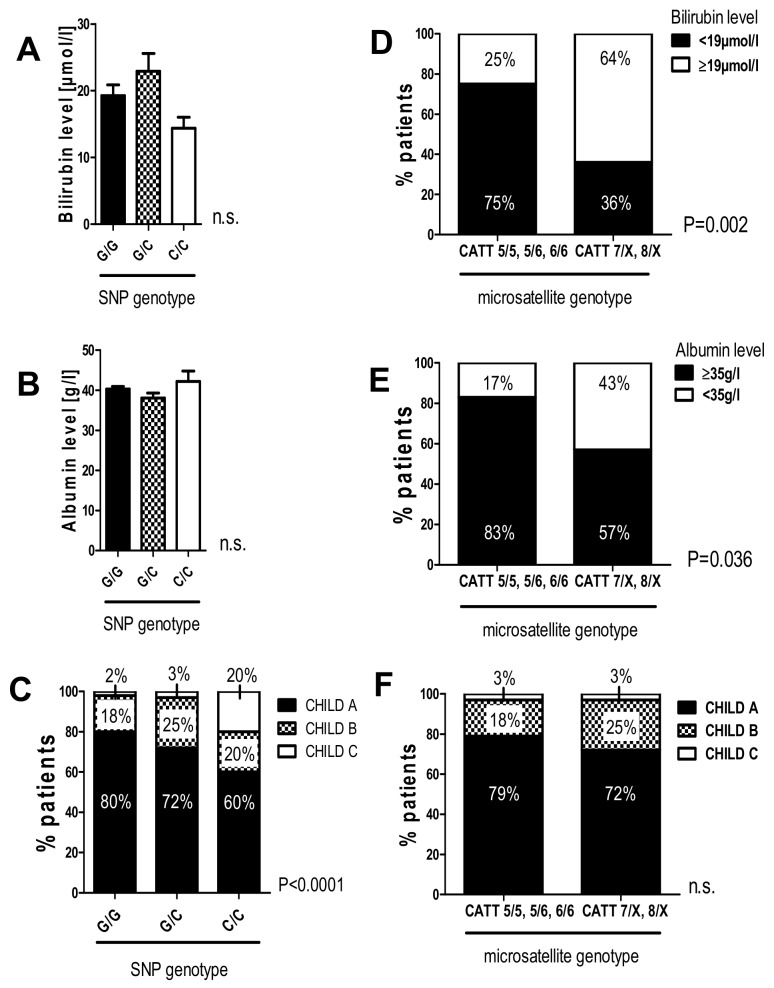
The G/C and C/C genotypes in the -173 G/C SNP and high microsatellite repeat counts in the -794 CATT_5–8_ are associated with worse liver function in patients with liver cirrhosis. Liver cirrhosis was diagnosed based on TE ≥ 12 kPa, if available, or morphological evaluation using a CAT scan. (**A**,**B**) Bilirubin and albumin serum levels did not differ between the three SNP genotype subgroups. (**C**) The proportion of patients with higher Child–Pugh stadium B and C was significantly higher within the patients´ subgroup with a G/C and C/C genotype according to the chi-square test (*p* < 0.0001). In the C/C genotype subgroup, 20% of patients have already progressed to Child–Pugh stadium C. (**D**,**E**) In the patients´ subgroup with higher microsatellite repeat numbers (CATT 7/X and 8/X), significantly higher percentages show bilirubin levels above and albumin levels below the reference cutoff respectively (cutoff for bilirubin ≥ 19 µmol/L; albumin < 35 g/L). (**F**) There was a tendency toward a higher Child–Pugh stadium within the CATT 7X; 8/X genotype subgroup. Data are expressed as the mean ± SEM (**A**,**B**) or contingency (**C**–**F**) and were considered significant, if *p* < 0.05 (*).

As shown in Figure 2, the minor alleles of the investigated polymorphisms were associated with lower fibrosis stages and liver stiffness; however, these tendencies only reached statistical significance in the haplotype analysis. Therefore, we went on to investigate whether these polymorphisms might correlate with liver function parameters and the clinical presentation of patients who already developed liver cirrhosis due to chronic HCV-infection. Therefore, we correlated the promoter polymorphisms with liver function tests, liver synthesis parameters, and the Child–Pugh stage as a composite score of liver function (Figure 3). Only patients with cirrhosis (liver stiffness ≥12 kPa or morphological evaluation using a CAT scan) were included, and bilirubin and albumin serum levels were dichotomized according to the lower/upper limits of normal (bilirubin < 19 µmol/L; albumin > 3.5 g/L).

Bilirubin and serum albumin did not differ between patients with the three SNP genotypes (Figure 3A,B). Analysis of the Child–Pugh stage, however, revealed a significantly higher proportion of patients with Child–Pugh stadium B or C in the C/C genotype subgroup (Child–Pugh B 20%, Child–Pugh C 20%) compared to the G/G subgroup (Child–Pugh B 18%, Child–Pugh C 2%; Figure 3C) (*p* < 0.0001 in Fisher’s exact test).

In the microsatellite genotype subgroups the proportion of patients with increased levels of bilirubin (≥19 µmol/L) and decreased levels of albumin (<35 g/L) was significantly higher in the patient subgroup with higher microsatellite repeats (CATT 7/X, 8/X) compared to those with lower microsatellite repeats (CATT 5/5, 5/6, 6/6; Figure 3D,E; *p* = 0.002 for bilirubin, *p* = 0.036 for albumin). Accordingly, this patient subgroup also showed a higher proportion of patients with Child–Pugh score B (Child–Pugh score B in the CATT 7/X, 8/X subgroup: 25% vs. 18% in the CATT 5/5, 5/6, 6/6 subgroup; Figure 3F); however, this effect was not significant.

### 2.8. The C/C Genotype in the -173 G/C SNP and High Microsatellite Repeat Counts in the -794 CATT_5–8_ Were Associated with a Higher Prevalence of Hepatocellular Carcinoma 

**Figure 4:**
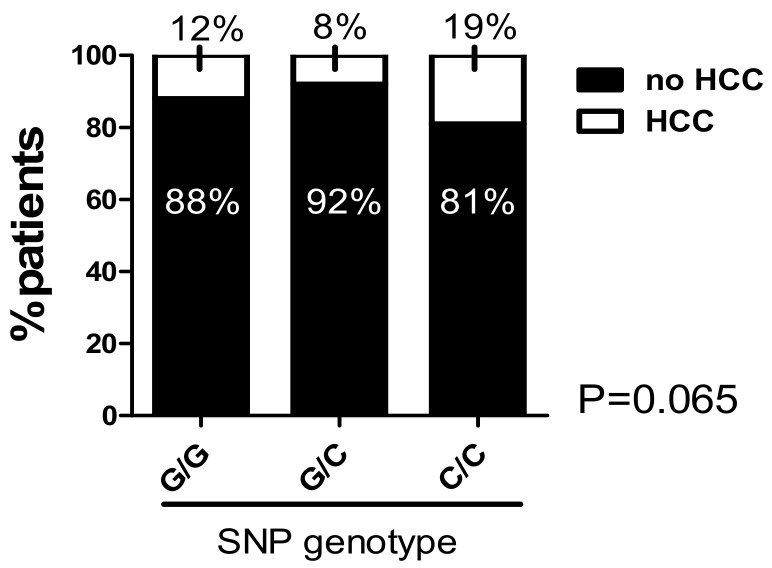
The C/C genotype in the -173 G/C SNP was associated with the prevalence of HCC: there is a strong tendency towards a higher HCC prevalence within the C/C genotype subgroup (*p* = 0.065). Data are expressed as contingencies.

In addition to the severity of HCV-induced liver fibrosis and cirrhosis, we evaluated the impact of the MIF promoter polymorphisms on the prevalence of HCC in patients with HCV-induced liver disease. Here, we see that for both minor genotypes, the prevalence of HCC was increased. However, for both minor genotypes, statistical analysis did not reach the level of significance, even if there was a strong tendency for the “C/C” SNP genotype (*p* = 0.065; Figure 4) (for microsatellite genotypes, see Supplementary Figure S2; *p* = 0.58).

## 3. Discussion

In this study we showed that the two promoter polymorphisms -173 G/C SNP and the -794 CATT_5–8_ microsatellite repeat in the MIF promoter, which are known to be associated with higher MIF serum levels, and exhibit a prognostic and clinical relevance in two large independent patient cohorts with HCV-induced chronic liver disease. Concerning the incidence and progression of liver fibrosis, the minor alleles (“C” in the -173 G/C SNP and higher repeat numbers “7/X, 8/X” in the microsatellite) of both polymorphisms were significantly associated with lower fibrosis stages in the first HCV 2008 cohort, and a tendency toward lower fibrosis stages was found in the second HCV 2018 cohort. However, in patients who had already progressed to cirrhosis, higher microsatellite repeats, as well as the “C/C” SNP genotype, were associated with significantly reduced liver function. Furthermore, the patient subgroup with the “C/C” SNP genotype showed a higher prevalence of hepatocellular carcinoma, even if this effect was not statistically significant.

Our study included two distinct, independent cohorts of Caucasian patients with chronic HCV-infection before the initiation of antiviral treatment. Based on both cohorts, we state here that the minor alleles of the investigated promoter polymorphisms were associated with lower fibrosis stages in HCV-induced fibrosis. Even if our analysis did not reach a level of significance in the comparison of minor versus major allele genotypes in the 2018 HCV cohort in contrast to the HCV 2008 cohort, the haplotype analysis in the HCV 2018 cohort revealed a statistically significant result regarding lower fibrosis levels in patients with “C/C” + “CATT 7/X, 8/X.” Based on this result, we adhere to our hypothesis that the minor allele genotypes were associated with a tendency toward lower fibrosis levels. Moreover, differences in the cohort characteristics and especially different methods to determine the level of fibrosis might have also been responsible for different results in the statistical analysis: In the 2008 cohort, the fibrosis stages and intrahepatic grading were determined using histological evaluation by experienced pathologists according to the Metavir score [24]. In the 2018 cohort, fibrosis stage was evaluated using non-invasive liver transient elastography (TE), which was first validated in patients who suffered from increased liver stiffness due to chronic HCV-infection [25]. The transient elastography represented a continuous variable in contrast to the histological fibrosis staging. Here, we used the cutoff of TE ≥9 kPa for advanced fibrosis (≥F2) and TE ≥ 12 kPa for F4 fibrosis, which was interpreted as liver cirrhosis as defined by Castera et al. in 2005 [26]. We assume that the TE was accompanied by a lower discriminatory power compared to histological staging, which made it more difficult to demarcate different fibrosis levels and to reveal significant differences. Next, to further baseline differences within the cohorts, this aspect might have been responsible for different statistical results.

MIF is expressed in several immune cells including monocytes/macrophages and T cells, as well as epithelial and endothelial cells [18]. MIF shows multifaceted functions that characterize its pro-inflammatory role relevant for immune cell recruitment and macrophage survival [18]. Accordingly, higher MIF serum levels have been shown to be associated with the severity of various inflammatory and autoimmune diseases [11,27]. In autoimmune hepatitis (AIH), serum MIF levels were increased compared to controls and associated with the necessity to receive steroid treatment [15]. This led us to hypothesize that MIF promoter polymorphisms influence chronic inflammation, thereby modulating fibrogenesis in our cohorts. As previously shown, the two investigated MIF promoter polymorphisms were associated with higher MIF serum levels [11,17,23]. Surprisingly, differences in liver fibrosis severity in the minor allele genotype groups were not an obvious consequence of increased intrahepatic inflammation as histological grading and ALT serum levels did not differ with respect to the genotypes. These results indicate that MIF may also convey hepatoprotective effects independent of inflammatory effects during fibrosis progression.

This notion is supported by preclinical data previously obtained by our group. In a mouse model of toxic liver fibrosis, we showed that MIF exhibits hepatoprotective effects attenuating fibrogenesis by directly interfering with the activation of hepatic stellate cells via the CD74/AMPK signaling pathway [28]. According to the murine data on the protective effect of MIF on fibrogenesis, our data also implicate a protective effect of minor allele genotypes that are supposed to be accompanied by increased MIF serum levels. We therefore argue that increased MIF serum levels due to higher transcriptional activity may explain the attenuated liver fibrosis in patients with C/C or CATT 7/X, 8/X genotypes.

While genotypes associated with higher MIF expression could be associated with decreased severity during the course of fibrogenesis, implicating a protective effect of MIF on fibrosis progression, the pictures seem to be inverted as soon as cirrhosis is established. Here, the minor frequent allele genotypes were significantly associated with worse liver function parameters and higher Child–Pugh scores, as well as characterized by a tendency toward the increased prevalence of HCC (not significant). However, interpretation of these data is limited due to the small sample size of minor allele patients and should be further evaluated in larger patient cohorts. Nevertheless, these data indicate that MIF exerts detrimental influences on the hepatic micromilieu and hepatocyte function/differentiation in cirrhosis and during hepatic carcinogenesis in contrast to its effect during fibrogenesis. In line with this, it has been shown that MIF is overexpressed in HCCs, and MIF serum levels correlate with tumor size and TNM (tumor/nodes/metastasis) stage [29]. As end-stage liver disease and hepatocarcinogenesis are associated with chronic intrahepatic and/or systemic inflammation, it is tempting to speculate that the pro-inflammatory effects of MIF, such as aggravating immune cell infiltration, might contribute to the increased incidence of complications such as decompensation or HCC development. However, this hypothesis warrants further mechanistical investigations.

Patients suffering from cirrhosis are at high risk for bacterial infections and sepsis due to a dysbalanced immune function [30]. Even in terms of compensated liver cirrhosis, and especially during acute decompensation, the composition of chemokines including MIF in the peripheral blood might be affected due to hepatic inflammation and systemic infection [31]. The clinical relevance of our study is to argue that the genotype determination of the investigated polymorphisms is a reasonable alternative compared to the determination of MIF serum levels as a prognostic parameter as the genetic variants were not influenced by systemic inflammation.

Concerning the frequency of the minor alleles of both investigated polymorphisms, our cohorts showed comparable frequencies of the “C” allele in the SNP and low repeat numbers in the microsatellite repeat with regard to the general population. For example, the frequency of the C/C genotype in the general population is 2%–10% [19,20], whereas in our cohorts, the frequency was 3% and 4%, respectively. Due to their low frequency in the general population, statistical analysis of the influence of these minor allele frequencies is challenging and further cohorts are needed to confirm our results.

In summary, here we show that the two promoter polymorphisms -173 G/C SNP and the -794 CATT_5–8_ microsatellite repeat are associated with the severity of liver disease in HCV infection in two independent patient cohorts. Regarding liver fibrogenesis the minor alleles of both polymorphisms show a prognostic accuracy for fibrosis progression; however, concerning severity of liver cirrhosis and hepatic carcinogenesis, both minor alleles seem to be disadvantageous. We argue that both polymorphisms imply MIF as a central player in the progression of fibrosis, cirrhosis, and the risk for hepatocellular carcinoma in a context-dependent manner. Rather than determining MIF serum levels, which are highly responsive to transient events, such as systemic inflammation, determination of the herein studied polymorphisms could represent novel and valid non-invasive marker concepts to predict the intra-individual course of disease in chronic HCV-infection and stratify patients at risk for complications in cirrhosis.

## 4. Materials and Methods 

### 4.1. Patients

Our study comprised patients´ data from two different patient cohorts. First, in 2008, second, until 2018, we collected data from overall 958 patients with chronic HCV infection. The patients were recruited from the outpatient departments of the universities in Berlin and Leipzig. Chronic HCV infection was diagnosed based on a positive anti-HCV assay and a positive HCV-RNA test. Additionally, the HCV genotype was determined as part of clinical routine. A baseline biopsy was performed before initiation of antiviral therapy in the 2008 patient cohort. Based on histological evaluation by experienced pathologists, fibrosis stages F1–F4 were allocated and inflammatory grading was ranked according to the Metavir score [24]. In the 2018 validation cohort, patients underwent transient elastography by Fibroscan^®^ before initiation of treatment and Fibroscan^®^ values were evaluated as follows: TE < 6 kPa (no fibrosis), TE 6–12 kPa (≥F2–3), TE ≥ 12 kPa (F4; liver cirrhosis). Grading of inflammatory activity was estimated based on levels of alaninaminotransferase (ALT) in patients´ serum. Alongside the ALT level, bilirubin and albumin levels were also determined in the serum of our patients. Furthermore, clinical criteria, such as the prevalence of HCC, as well as the Child–Pugh score, were documented at the time of inclusion.

### 4.2. Ethical Approval

The study protocol was approved by the medical ethics committees (Berlin: 205/2002 from Ethics Committee University Hospital Charité Berlin; date of approval: 19 November 2002; Leipzig: 252/18-lk from Ethical Commission University Hospital Leipzig; date of approval: 31 July 2018) and informed consent was obtained from patients prior to inclusion.

### 4.3. Determination of MIF Promoter Polymorphisms -173 G/C (rs755622) and -794 CATT_5–8_ (rs5844572)

Genomic DNA was extracted from peripheral whole blood patient samples using a customized kit NucleoSpin^®^ Blood (Macherey Nagel, Düren, Germany). Genotyping of patients for the single nucleotide polymorphism (SNP) rs755622 (-173 G/C) was performed with genomic DNA with 5′-endonuclease (TaqMan, applied biosystems, Darmstadt, Germany) assays. For rs755622 SNP primer sequences were TTTCTAGCCGCCAAGTGGAGAACAG[C/G]TTGGAGCGGTGCGCCGGGCTTAGCG. Fluorescence labeled primers and assay conditions were obtained from Applied Biosystems. Fluorescence was measured using an ABI PRISM 7000 Sequence Detection System (Applied Biosystems, Darmstadt, Germany). The *-173 G/C SNP* genotypes were classified as G/G (homozygous for the major allele), and G/C (heterozygous) or C/C (homozygous for the minor allele).

Fragment analysis to determine the repeat length of the tetra nucleotide marker rs5844572 (-*794 CATT_5–8_*) was performed using PCR with sense [GACAGGACCTCCCTGGAAAT] and antisense [TCATAGAGCCCTTGGTGAAT] primers; PCR conditions are available on request. PCR products were run on an AB3130 genetic analyzer (Applied Biosystems), and electrophoresis results were analyzed with the GeneMapper^®^ Software 5 (Applied Biosystems).

### 4.4. Statistical Analysis

Statistical analysis was performed with GraphPad software version 5.01 (GraphPad, San Diego, CA, USA) and IBM SPSS Statistics 25 (IBM Corp., Armonk, NY, USA). Allele frequency difference was analyzed and complemented with linear-by-linear association for the SNP genotypes (Table 2, Table 3, Table 5, Table 6). A one-way ANOVA with the Kruskal–Wallis test was used when comparing more than two different groups. Data is given here as median ± range (Figure 1A,B) or mean ± SEM (Figure 2A,D; Figure 3A,B). Continuous variables were compared using a two-sided Student’s *t*-test with Welch’s correction in case of unequal variances as mean ± standard error of the mean (Figure 2C). Contingency data were evaluated using Fisher´s exact test (Figure 1C,D; Figure 3D–F, Figure 4) or chi-square test (Figure 1E,F; Figure 2B,E; Figure 3C) and was complemented with a linear-by-linear association (Figure 2B). A *p*-value < 0.05 was considered significant.

## Figures and Tables

**Table 1:** Cohort characteristics of the HCV 2008 cohort including a histological evaluation of the fibrosis stage (“staging”) and intrahepatic inflammatory activity (“grading”) according to the Metavir score [24]. Only patients with a Metavir staging of ≥F0.5 were included in the study.

	*n* = 473
Age (years)	54 (26–87)
Metavir Staging	*n* (%)
F0.5 F1–F1.5 F2–F2.5 F3–F3.5 F4	5 (1) 193 (41) 168 (36) 67 (14) 40 (8)
**Metavir Grading**	***n* (%)**
0.5 1–1.5 2–2.5 3–3.5 4	8 (2) 210 (44) 227 (48) 26 (5.5) 2 (0.5)

**Table 2:** SNP genotype frequencies in the HCV 2008 cohort as observed in patients with fibrosis (stage >F0) when stratified for the presence of significant fibrosis (stage ≥F2).

	SNP -173 G/C (rs755622)				Allele Frequency Difference		Linear-by-Linear Association	
Phenotype (n)	G/G	G/C	C/C	Freq. (C)	Odds Ratio (95% CI)	*p*-value	X²	*p*-value
<F2 (198)	120 (61%)	69 (35%)	9 (5%)	0.20	0.66 (0.48–0.93)	0.015	6.07	0.014
≥F2 (273)	192 (70%)	76 (28%)	5 (2%)	0.16				

**Table 3:** Microsatellite genotype frequencies in the HCV 2008 cohort as observed in patients with fibrosis (stage > F0) when stratified for the presence of significant fibrosis (stage ≥ F2).

	Microsatellite CATT_5–8_ (rs5844572)			
Phenotype (n)	CATT 5/5, 5/5, 6/6	CATT 7/X, 8/X	Odds Ratio (95% CI)	*p*-value
<F2 (197)	138 (70%)	59 (30%)	0.64 (0.42–0.97)	0.04
≥F2 (271)	213 (79%)	58 (21%)		

**Table 4:** Cohort characteristics for HCV cohort 2018 including non-invasive evaluation of fibrosis stage using transient elastography (TE), as well as clinical features as available as Child Pugh stadium, prevalence of cirrhosis, and hepatocellular carcinoma. Furthermore, the HCV genotype was determined in this cohort.

	***n* = 443**
Age (years)	52 (18–86)
Gender	*n* (%)
Male Female	214 (48) 229 (52)
Transient Elastography (Fibroscan^®^)	*n* (%)
<6 kPa 6–12 kPa ≥12 kPa	144 (38) 120 (32) 113 (30)
Liver Cirrhosis	*n* (%)
No Yes	290 (66) 152 (34)
Child–Pugh Stadium	*n* (%)
A B C	93 (76) 24 (20) 4 (3)
HCC	*n* (%)
No Yes	394 (89) 49 (11)
Liver transplant	*n* (%)
No Yes	417 (94) 26 (6)
HCV Genotype	*n* (%)
1 2 3 4	350 (83) 7 (2) 50 (12) 13 (3)

**Table 5:** SNP genotype frequencies in the HCV 2018 cohort as observed in the two patient subgroups with advanced versus non-advanced fibrosis stages based on the transient elastography (TE) cutoff of 9 kPa.

	SNP -173 G/C (rs755622)				Allele Frequency Difference		Linear-by-Linear Association	
Phenotype (n)	G/G	G/C	C/C	Freq. (C)	Odds Ratio (95% CI)	*p*-value	X²	*p*-value
TE < 9 kPa (228)	149 (65%)	72 (31%)	7 (3%)	0.19	0.73 (0.49–1.08)	0.11	2.49	0.14
TE ≥ 9 kPa (149)	110 (74%)	35 (23%)	4 (3%)	0.14				

**Table 6:** Microsatellite genotype frequencies in the HCV 2018 cohort as observed in the two patient subgroups with high or low fibrosis stages based on the TE cutoff ≥12 kPa.

	Microsatellite CATT_5–8_ (rs5844572)			
Phenotype (n)	CATT 5/5, 5/5, 6/6	CATT 7/X, 8/X	Odds Ratio (95% CI)	*p*-value
TE <9 kPa (212)	159 (75%)	53 (25%)	0.89 (0.53–1.47)	0.70
TE ≥9 kPa (136)	105 (77%)	31 (23%)		

## Supplementary Materials

Supplementary figures can be found at https://www.mdpi.com/1422-0067/20/15/3753/s1.

## Abbreviations

MIF	Macrophage migration inhibitory factor
SNP	Single nucleotide polymorphism
HCC	Hepatocellular carcinoma
FS	Fibroscan^®^
DAA	Direct-acting antivirals
ALT	Alaninaminotransferase
SVR	Sustained virological response
AIH	Autoimmune hepatitis
TE	Transient elastography
