# Supplementary material for: Genetic Variants in the Promoter Region of the Macrophage Migration Inhibitory Factor are Associated with the Severity of Hepatitis C Virus-Induced Liver Fibrosis
Source: Int J Mol Sci. 2019 Jul 31;20(15):3753. doi: 10.3390/ijms20153753 (PMC6696142; doi:10.3390/ijms20153753)
Supplement: Supplementary file 1 [file ijms-20-03753-s001.pdf]

Supplementary Figures:

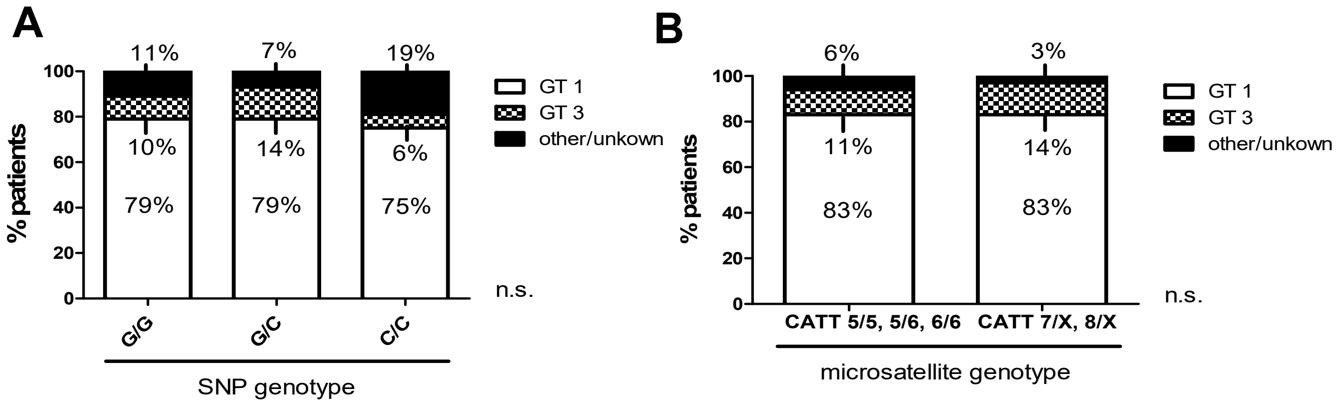

Figure S1. HCV genotype frequency does not differ significantly in SNP and microsatellite genotype subgroups of the HCV 2018 cohort. **(A)** The prevalence of the HCV genotype is depicted in the three SNP genotype subgroups "G/G", "G/C" and "C/C". Here no significant difference concerning HCV genotype 1 ("GT 1"), 3 ("GT 3") or patients infected with HCV genotype 2 or 4 or unkown HCV genotype ("other/unkown") is observed. **(B)** The prevalence of the HCV genotype is depicted in the two microsatellite genotype subgroups "CATT 5/5, 5/6, 6/6" and "CATT 7/X, 8/X". Here no significant difference concerning the HCV genotype 1 ("GT 1"), 3 ("GT 3") or patients infected with HCV genotype 2 or 4 or unkown HCV genotype ("other/unkown") is observed.

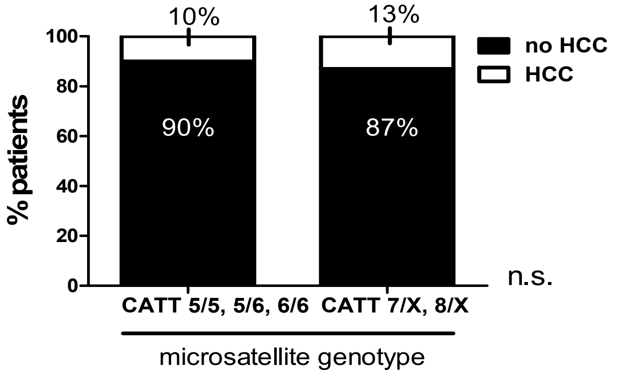

Figure S2. High microsatellite repeat counts in the -794 $CATT_{5\text{-}8}$ are not associated with prevalence of HCC: The HCC prevalence does not differ significantly between the two microsatellite repeat genotype subgroups. Data are expressed as contingency.
